# Electrodeposition of the MnO_2_ on the Ag/Au Core–Shell Nanowire and Its Application to the Flexible Supercapacitor

**DOI:** 10.3390/ma14143934

**Published:** 2021-07-14

**Authors:** Wonbin Seo, Dongwoo Kim, Shihyeong Kim, Habeom Lee

**Affiliations:** 1 School of Mechanical Engineering, Pusan National University, 2, Busandaehag-ro, 63 Beon-gil, Geumjeong-gu, Busan 46241, Chungcheongnam-do, Korea; wonvin0506@pusan.ac.kr (W.S.); 201521135@pusan.ac.kr (D.K.); 2 Technical Textile & Materials R&D Group, Korea Institute of Industrial Technology, Ansan 15588, Gyeonggi-do, Korea; shk@kitech.re.kr

**Keywords:** flexible supercapacitor, Ag nanowire, Ag/Au core–shell nanowire, MnO_2_ supercapacitor

## Abstract

Supercapacitors have received considerable attention as energy storage devices owing to their high power density, fast charge/discharge rate, and long cyclic life. Especially with an increasing demand for flexible and wearable devices, research on flexible supercapacitors has surged in recent years. The silver nanowire (Ag NW) network has been used as a flexible electrode owing to its excellent mechanical and electrical properties; however, its use as an electrode for flexible supercapacitors has been limited due to insufficient electrochemical stability. In this study, we proposed a method to resolve this issue. We employed a solution process that enabled the coating of the surface of Ag NW by a thin Au shell of ≈ 5 nm thickness, which significantly improved the electrochemical stability of the Ag NW network electrodes. Furthermore, we confirmed for the first time that MnO_2_, which is one of the most widely used capacitive materials, can be directly electroplated on the AACS NW network electrode. Finally, we fabricated a high-performance and flexible solid-state supercapacitor using the suggested Ag/Au/MnO_2_ core–shell NW network electrodes.

## 1. Introduction

Supercapacitors are energy storage devices that exhibit a high power density, fast charge/discharge rate, and long cyclic life; thus, they have received considerable research interest [1,2,3,4]. In recent years, the growing interest in flexible and wearable devices has motivated active research on flexible supercapacitors [5,6,7,8]. The most basic components of energy storage devices such as supercapacitors are a current collector, a passage for electrons, and an active layer capable of storing electrical energy. In some cases of electrical double-layer capacitors (EDLCs), one electrode simultaneously serves as both the current collector and the active layer. However, in the most cases of pseudocapacitors, which can achieve a larger storage capacity based on reversible redox reactions, the current collector and active layer are clearly distinguished. Therefore, the fabrication of a flexible supercapacitor with high storage capacity requires the flexible electrode as a current collector and a technique capable of applying an active layer on the electrode.

Among the various materials for flexible electrodes, the Ag NW network is one of the most promising candidates because of the properties such as transparency [9], flexibility [10,11], high electrical conductivity [12], and mature fabrication techniques [13]. However, it is difficult to directly use the Ag NW network as a flexible current collector for supercapacitors. The energy storage performance of a supercapacitor is greatly affected by the operating voltage range; silver exhibits irreversible redox reactions and loses its electrical conductivity within the typical operating voltage range of an aqueous electrolyte-based supercapacitor [14,15]. We recently reported that the electrochemical stability of the Ag NW network electrode could be improved using a thin protective Au layer. The suggested Ag/Au core–shell NW (AACS NW) network showed cycle stability within the typical voltage range of aqueous electrolyte-based EDLCs (0–0.8 V) [16]. Pseudocapacitors have a higher energy storage capacity than EDLCs due to their energy storage mechanisms based on reversible redox reactions [17,18,19] in the active layer. MnO_2_ is one of the most widely studied transition metal oxides for pseudocapacitors because of its low cost, natural abundance, large theoretical capacity, and low toxicity [20,21]. As MnO_2_ has low electrical conductivity (10^−5^–10^−6^ S cm^−1^) [22], it needs another conductive layer to constitute the supercapacitor electrode. The most common method to combine MnO_2_ with the current collector is the electroplating process. However, there are some limitations to depositing MnO_2_ on the Ag NW network using the electroplating process, because the electrochemically unstable Ag NWs are easily damaged during the electroplating process. Even if MnO_2_ can be coated on the Ag NWs, the electrode still cannot operate in the typical voltage range of supercapacitors for the same reason.

Herein, we confirmed that it is possible to electroplate a MnO_2_ active layer on the Ag NW network electrode by enhancing the electrochemical stability of the Ag NW network electrode with the Au protecting layer on the surface of the Ag NW. The Au-protecting layer was deposited on the surface of the Ag NW through a galvanic replacement-free solution process [16]. We demonstrated a flexible pseudocapacitor with Ag/Au/MnO_2_ core–shell NWs and examined its mechanical and electrochemical performances. The suggested supercapacitor showed no decrease in the capacitive performances even under the 5.5 mm bending radius, and its areal capacitance was reserved over 92% even during the 1000 times repeated bending cycles. The flexibility and the mechanical stability of the supercapacitor resulted from the conserved properties of the Ag NW network electrode. It is expected that the method proposed in this study for improving the electrochemical stability of Ag NW network electrodes will further expand the application field of Ag NW network-based flexible electrodes to flexible energy devices, including supercapacitors and batteries.

## 2. Materials and Methods

### 2.1. Preparation of Ag/Au Core–Shell NW

Polyvinylpyrrolidone (PVP), L-ascorbic acid (AA), gold(III) chloride hydrate (HAuCl_4_), and sodium hydroxide (NaOH) were obtained from Sigma-Aldrich. The Ag NW aqueous solution was provided by N&B Co. and used without further purification. First, 100 μL of a 0.5 wt % Ag NW solution was added to an aqueous solution of PVP (1 mM, 20 mL), AA (0.1 M, 5 mL), and NaOH (0.2 M, 5 mL). During vigorous stirring of the solution, we slowly injected the HAuCl_4_ aqueous solution (0.15 mM, 12 mL) using a motorized syringe pump at an injection rate of 40 μL/min. After the injection process, we collected the resultant NWs using a centrifuge. In detail, the solution was transferred to a conical tube and centrifuged at 5000 rpm for 10 min. After removing solvents, the precipitated NWs were dispersed in DI water, and the solution was centrifuged again at the same condition. Finally, the collected NWs were dispersed in DI water (2 mL) and ethanol (18 mL) to complete the preparation of the AACS NW solution.

### 2.2. Preparation of the Ag NW and Ag/Au Core–Shell NW Network Electrodes

We prepared the NW network electrodes through the vacuum filtration and transfer method [16]. A nylon filter (0.5 μm pore size), a PTFE filter (0.5 μm pore size), a glass funnel, and a vacuum pump were connected to a filtering flask. Then, 3–7 mL of the NW solution was poured into the funnel after operating the vacuum pump. The filtered NWs, forming an interconnected network on the PTFE filter, were transferred to a target substrate such as polyethylene terephthalate (PET) film and glass. In the transfer process, the funnel was gently removed while operating the vacuum pump, and the plasma-treated target substrate was placed on the PTFE filter. Moderate pressure was applied to the substrate manually for a few minutes. Then, the vacuum pump was stopped, and the vacuum in the flask was released to detach the target substrate from the filtering flask. Finally, we gently removed the nylon and PTFE filters from the target substrate to leave an electrically conductive NW network on the PET film. 

### 2.3. Electroplating of the MnO_2_ on the Ag/Au Core–Shell NW Network Electrode

The NW network electrode was cut into the desired shape and size for the MnO_2_ electroplating. We connected the copper tape to one side of the NW network electrode with silver paste. Then, the MnO_2_ was electroplated on the NW network by a three electrode method with a constant DC voltage of 1.5 V. A mixture of Mn(NO_3_)_2_ (20 mM, 50 mL) and NaNO_3_ (100 mM, 50 mL) aqueous solution was used as the plating solution. In this electroplating process, we controlled the coating amount of MnO_2_ by adjusting the coating time.

### 2.4. Preparation of an All-Solid State Supercapacitor

Polyvinyl alcohol (PVA) and LiClO_4_ were obtained from Sigma-Aldrich and used without further purification. A gel electrolyte was prepared by dissolving 2 g of PVA and 0.67 g of LiClO_4_ in 20 mL of DI water at 85 °C. After cooling, we immersed the Ag/Au/MnO_2_ core–shell NW network electrodes in the gel electrolyte for 1 min. Then, we kept the electrolyte deposited electrodes at room temperature for 4 h to remove residual moisture from the electrolyte. Subsequently, we stuck a pair of gel electrolyte-coated electrodes on top of each other to fabricate a solid-state supercapacitor. Here, the gel electrolyte layers act as an adhesive.

### 2.5. Material Characterizations and Electrochemical Performance Measurements 

We observed the surface morphologies of the materials using field-emission scanning electron microscopy (FE-SEM, Hitachi S-4700, Hitachi, Ibaraki, Japan). For more detailed material analyses, transmission electron microscopy (TEM, TALOS F200X, Waltham, MA, USA), X-ray diffraction (XRD, D8 Advance, Billerica, MA, USA), and X-ray photoelectron spectroscope (XPS, AXIS-HSi, Manchester, U.K.) were used. The electrochemical performances of the half-cell electrodes were obtained through a three-electrode method using Pt mesh as a counter electrode and Ag/AgCl (3.5 M KCl) electrode as a reference electrode in a 1.0 M Na_2_SO_4_ aqueous solution. The electrochemical performances of the solid-state supercapacitor were measured in a two-electrode method. We used a potentiostat (Versa STAT 3, Berwyn, PA, USA) for entire electrochemical analysis.

## 3. Results and Discussion

### 3.1. Preparation of the AACS NW Electrodes 

**Figure 1:**
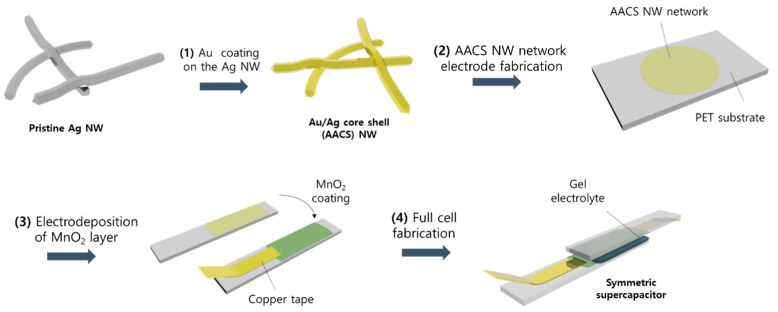
Schematic illustration of Ag/Au/MnO_2_ core–shell NW network electrodes and solid state.

The entire process of fabricating a flexible supercapacitor based on the electrochemical stability enhanced Ag NW network is shown in Figure 1. The first step is to prepare the AACS NWs. The AACS NWs were prepared by an Au layer coating on the surface of commercial Ag NWs (20 nm in diameter, 20 μm in length). The coating process suggested and used in this research is an all-solution process that can form a thin Au layer on the Ag NW surface without galvanic replacement, which causes the destruction of the Ag NW. This method is a straightforward process involving a slow injection of the Au precursor solution into a vigorously stirred Ag NWs aqueous solution containing capping, reducing, and pH-increasing agents. Compared to other Au coating processes based on physical vapor deposition, this method has the advantage of minimizing the waste of Au, which is considered as the noblest metal, because the Au atoms are selectively deposited only on the target material, the Ag NW surface. In this Au coating process, two main factors strongly affect the quality of the resulting core–shell NWs. The two factors are the pH of the reaction solution and the injection rate of the Au precursor. The adequately high pH of the reaction solution prevents galvanic replacement between the Ag and the Au [23]. When a galvanic replacement reaction occurs, it is impossible to form an even Au layer on the Ag NW surface, as shown in Figure S1a. In addition, even if the galvanic replacement reaction is prevented by adjusting the pH, it is difficult to fabricate the NW network electrode if aggregation occurs between NWs during the Au coating process. It is hard to separate the NWs once the NWs agglomerate each other, as shown in Figure S1b. To avoid the aggregation between the NWs, we injected the Au precursor solution at a sufficiently slow rate of 50 μL/min or less. More details of the experimental procedure, such as the materials, concentration of each solution, and injection rate are included in the Materials and Methods section.

**Figure 2:**
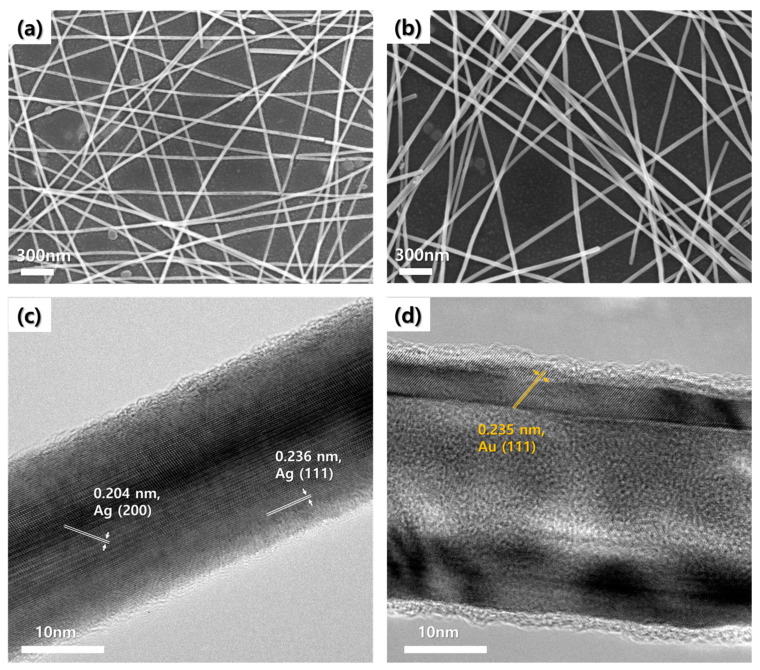
SEM and TEM images of Ag NWs and AACS NWs. (**a**) SEM image of the Ag NW network. (**b**) SEM image of the AACS NW network. (**c**) TEM image of a pristine Ag NW. (**d**) TEM image of an AACS NW.

We used the vacuum filtration and transfer method to form an AACS NW network on the PET substrate. As a control sample, the Ag NW network electrode was prepared through the same method. Figure 2a,b show the SEM images of the Ag NW and AACS NW network electrodes, respectively. In both cases, the NWs with high aspect ratio formed the inter-connected network structures. Furthermore, we performed TEM analysis to investigate the AACS NWs more closely. Figure 2c,d show the TEM images of the pristine Ag NW and AACS NW. In Figure 2c, we observed the definite and ordinary lattice plane and calculated the plane distance using image analysis software. The calculated lattice distances (0.236 nm, 0.204 nm) correspond to the (111) plane and (200) plane of Ag crystal structure (2.359 $\dot{A}$, 2.044 $\dot{A}$, PDF4-00-004-0783). In the TEM image of the AACS NW presented in Figure 2d, we observed a contrast in brightness between the central region and the edges of the NW. It means that the materials in the two areas have different crystal structures or different crystal orientation. We calculated the lattice plane distance of the surface region to 0.235 nm. The value corresponds to the (111) plane of the Au crystal structure (2.354 $\dot{A}$, PDF4-00-066-0091). The results explain that the dark contrast at both sides of the AACS NW was derived from the Au layer on the Ag NW surface. In addition, we confirmed that the Au layer coated on the Ag NW surface had a thickness of ≈5 nm by comparing the diameter between the pristine Ag NW (21 ± 3 nm) and the AACS NW (31 ± 3 nm). The NWs’ diameters were measured through the TEM analysis, as shown in Figure S2. TEM-EDX mapping images in Figure S3 intuitively show the AACS NWs in which the Au shells cover the Ag NWs. For further examination, we measured the TEM-EDX line profile following the yellow lines of the TEM images of the Ag NW and the AACS NW presented in Figure S4a,c, respectively. Figure S4b,d show that the dark contrast at both edges of the AACS NW in Figure 2d resulted from the Au layer on the Ag NW surface.

### 3.2. Properties of the AACS NW electrode

**Figure 3:**
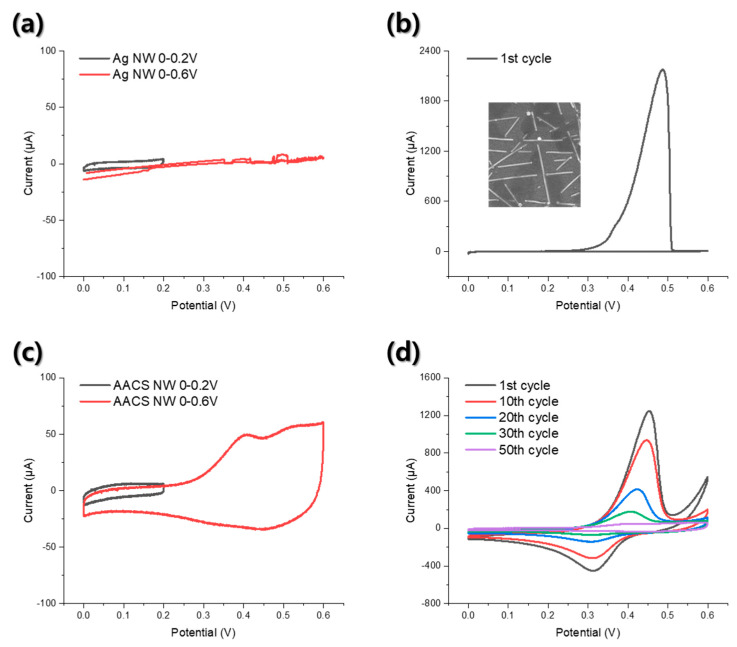
Electrochemical properties of the Ag NW and the AACS NW network electrode. (**a**) CV curves for the Ag NW electrode. The black line shows the CV curve in the 0−0.2 V scan range, and the red line shows the CV curve of the second cycle in the 0−0.6 V scan range. (**b**) CV curve of the first CV cycle in the 0−0.6 V scan range for the Ag NW electrode. The inset figure shows the SEM image of the Ag NW network after the CV cycles in the 0−0.6 V scan range. (**c**) CV curves for AACS NW electrode. The black line shows the CV curve in the 0−0.2 V scan range, and the red line shows the CV curve of the 50th CV cycle in the 0−0.6 V scan range. (**d**) CV curves for AACS NW electrode from the 1st to the 50th CV cycles.

To inspect the mechanical flexibility of the AACS NW network electrode as a flexible current collector for the supercapacitor, we performed repeated bending tests while monitoring the change of the electrical resistance of the Ag NW and AACS NW network electrodes. As shown in Figure S5, both electrodes maintained their electrical conductivity without significant changes in electrical resistance during the 1000 times repeated bending test with a bending radius of 5.5 mm. A more critical part here is whether we can improve the electrochemical stability of the Ag NW networks electrode through the Au shell. To examine the electrochemical stability enhancement through the Au layer, we performed CV tests using 1M Na_2_SO_4_ aqueous solution as an electrolyte, Pt mesh as a counter electrode, and Ag/AgCl (3.5 M KCl) electrode as a reference electrode. As shown in Figure 3a, the Ag NW electrode showed a rectangular-shaped CV curve in the 0–0.2 V range, and there were no apparent oxidation or reduction peaks. However, when we extended the voltage scan range to 0–0.6 V, the Ag NW network electrode showed an irregular and unstable current value, as displayed by the red line. The CV curve was obtained from the second CV cycle. The first CV cycle of the Ag NW electrode, as shown in Figure 3b, explains the reason for the electrical short. During the forward voltage scanning, a clear current peak was observed in the range of 0.4 to 0.5 V, resulting from the oxidation of the Ag [14]. However, in the subsequent backward voltage scanning, no corresponding negative current peak (reduction peak) was observed, indicating that irreversible oxidation occurred in the Ag NW network electrode. The inset image in Figure 3b shows the Ag NW network SEM image after the CV test in the 0–0.6 V range. From the SEM image, we observed that the individual NWs were destructed, and the network disconnected due to the irreversible oxidation of the Ag. Figure 3c shows the CV curves of the AACS NW network electrode. In the 0–0.2 V range, the AACS NW electrode exhibited the rectangular-shaped CV curve, as shown in the black line. When we extended the voltage scan range to 0–0.6 V, the AACS NW electrode showed oxidation peaks such as the Ag NW electrode, as shown in Figure 3d, and the peak potential was the same as that of the Ag NW electrode. Thus, it can be said that the oxidation peak resulted from the Ag NW rather than the Au shell. However, in contrast to the Ag NW electrode, the AACS NW electrode showed reduction peaks corresponding to oxidation peaks. Unlike the Ag NW electrode, even if some Ag atoms were ionized since the Au shell conserved the electrical conductivity of the electrode, some Ag ions nearby the electrode were reduced back to Ag during the backward voltage scanning, resulting in the reduction peaks. In addition, the current values of the redox peaks gradually decreased and eventually disappeared after the 50th CV cycle. The CV curve obtained from the 50th CV cycle is presented as the red line in Figure 3c. As a result, the AACS NW electrode did not show abrupt electrical short and exhibited a stable CV curve even after the repeated CV cycles. Through this, we confirmed that the Au shell coated on the Ag NWs surface improved the electrochemical stability of the Ag NW network electrode and provided the additional electrical path to the electrode, allowing it to be used as a supercapacitor current collector in a wider voltage range.

### 3.3. Electroplating of MnO_2_ on the AACS NW Network Electrode

Improving the electrochemical stability of the Ag NW network electrode by the Au protecting layer could be confirmed again through the electroplating process of MnO_2_. We attempted the MnO_2_ coating on the Ag NW and the AACS NW network electrodes using the same electroplating process. The input voltage was kept at 1.5 V for 1 min, and a mixture of Mn(NO_3_)_2_ (20 mM, 50 mL) aqueous solution and NaNO_3_ (100 mM, 50 mL) aqueous solution was used as a plating solution [24]. We examined the changes in the appearance and the electrical conductivity of the electrodes before and after the electroplating process, as shown in Figure S6a. The Ag NW electrode showed no noticeable difference in appearance during the electroplating process but lost its electrical conductivity. The loss of electrical conductivity of the Ag NW electrode during the electroplating process is due to the irreversible oxidation reaction of the Ag as confirmed by the electrochemical stability test through CV analysis. However, the AACS NW electrode maintained its electrical conductivity with a slight increase in electrical resistance, and the AACS NW electrode showed distinct changes in its transparency and color after the same electroplating process. The light transmittance of the AACS NW electrode was sharply decreased after the electroplating. In contrast, the optical transmittance of the Ag NW electrode slightly increased after the electroplating, as can be seen in Figure S6b. In addition, the digital image of the Ag NW, the AACS NW, and the 1-min electroplated AACS NW electrode in Figure S6c shows the color change of the AACS NW electrode during the electroplating. Therefore, we could expect that the increase in the electrical resistance of the AACS NW electrode after the electroplating process resulted from the MnO_2_ layer with relatively low electrical conductivity on the surface of the AACS NWs. Figure S6d shows the CV curves for the Ag NW and the AACS NW network electrode after the electroplating process. In the Ag NW electrode, no CV characteristics were observed, whereas in the case of the AACS NW, a rectangular-shaped stable CV curve was observed. It means that through the electroplating process, a capacitive active layer was formed on the AACS NW network electrode.

**Figure 4:**
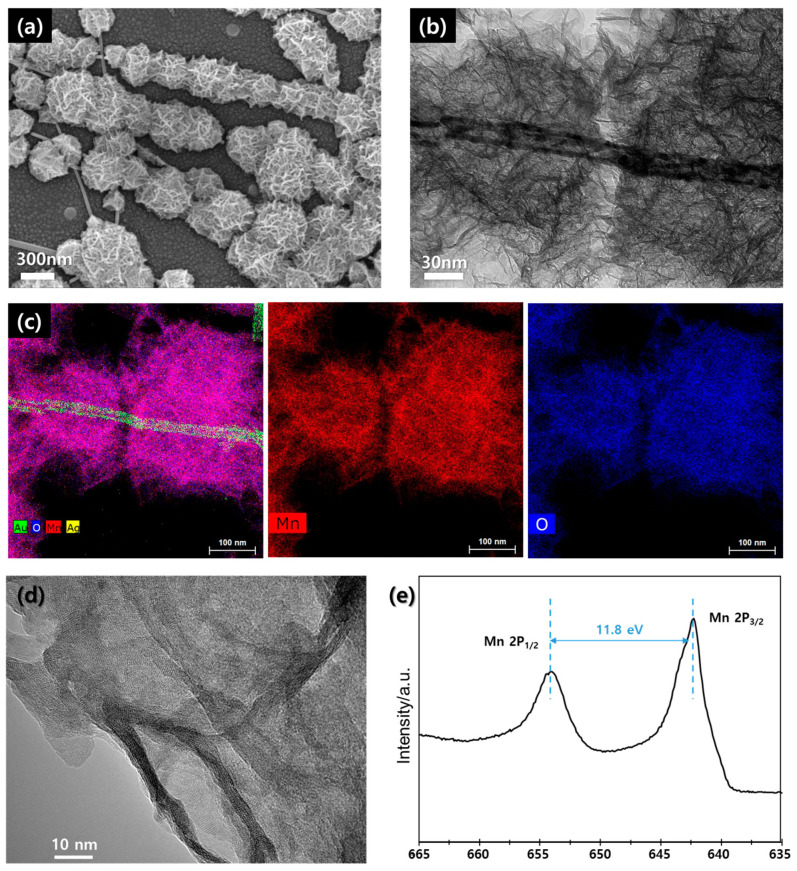
(**a**) SEM image. (**b**) TEM image. (**c**) TEM-EDX mapping image. (**d**) HRTEM image. (**e**) XPS analysis result of the electro-plated material on the AACS NW.

Figure 4a shows the SEM image of the electroplated layer on the AACS NWs. Porous nanostructures were formed along the AACS NWs. We performed the TEM analysis to examine the material’s properties and presented the results in Figure 4b–d. We fabricated the AACS NW network on a glass substrate rather than a PET substrate, performed electroplating, and scraped the resultant material with a doctor blade to prepare a TEM sample. Figure 4c shows the TEM-EDX mapping images corresponding to the TEM image shown in Figure 4b. It can be seen that the structure formed on the surface of AACS NW is composed of Mn and O. However, as can be seen from the HRTEM image presented in Figure 4d, the electroplated layer did not show the obvious crystal structure. Likewise, in the XRD analysis presented in Figure S7, we could not observe any peaks for the crystal structure of the MnO_2_. Rather, we only obtained the signals for Au and Ag from the XRD analysis. So, through X-ray photoelectron spectroscopy (XPS) analysis, we tried to examine the electroplated materials’ elemental composition more precisely. We presented the XPS spectra for Mn-2P in Figure 4e. The separation of spin energy between Mn 2P^1/2^ (654.2 eV) and Mn 2P^3/2^ (642.4 eV) was measured to 11.8 eV, and it means that the electroplated materials are MnO_2_ [25]. As a result, owing to the thin Au protecting layer on the Ag NW surface, we could successfully fabricate the Ag/Au/MnO_2_ core–shell NW network electrode through an electroplating process.

### 3.4. Electrochemical Performances of the Ag/Au/MnO_2_ Core–Shell NW Electrode

To examine the performance of the Au/Ag/MnO_2_ core–shell NW network electrode as a supercapacitor, we studied the capacitance dependency on the areal density of the AACS NWs first. In the vacuum filtration and transfer method, we controlled the AACS NW areal density by fixing the concentration of the AACS NW solution at 0.025 mg/mL and changing the volume of the solution to be filtered from 3 to 7 mL. Then, the MnO_2_ layer was electroplated on each electrode under the same conditions for 2 min. Then, the cyclic voltammetry was measured using a three-electrode method with Na_2_SO_4_ aqueous solution. At this time, the voltage scan rate was set as 100 mV/s. 

**Figure 5:**
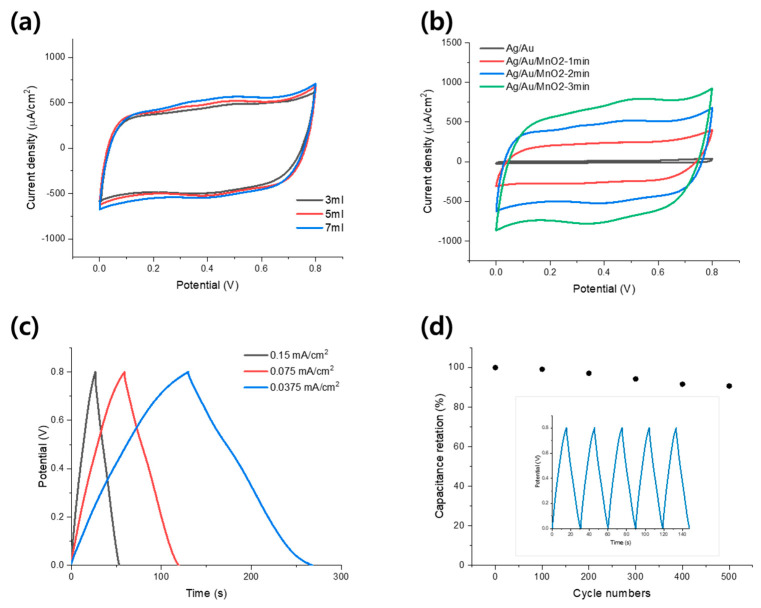
Electrochemical performances of the Ag/Au/MnO_2_ core−shell NW network electrodes. (**a**) CV curves of Ag/Au/MnO_2_ core−shell NW electrodes with different areal densities of AACS NW at 100 mV/s voltage scan rate. (**b**) CV curves of the electrodes prepared through various coating times (from 0 to 3 min). Here, the CV curve of the AACS NW (black line) was obtained from the 50th CV cycle (same data with the Figure 3c). (**c**) Charge−discharge curves of the Ag/Au/MnO_2_ core−shell NW electrode with different current densities. (**d**) Cyclic stability of the Ag/Au/MnO_2_ core−shell NW electrode in a continuous charge−discharge test at a 0.2 mA cm^−2^ current density.

The results are presented in Figure 5a; all three cases show almost rectangular-shaped CV curves. It shows that the Ag/Au/MnO_2_ core–shell NW electrodes had good capacitive behavior with rapid charge and discharge over the entire voltage scan range. As the NW areal density increased, the specific current value of the CV curve tended to increase slightly, but the difference was not very large. Therefore, we concluded that the capacitive performance of the Ag/Au/MnO_2_ core–shell NW electrode does not change significantly depending on the areal density of the AACS NW. In the following experiments, we used AACS NW electrodes made from a 5 mL solution. Next, the changes in the capacitive performance of the electrode according to the coating amount of MnO_2_ were examined. The amount of MnO_2_ was adjusted by controlling the electroplating time from 0 to 3 min. Based on the current change during the electroplating process, as shown in Figure S8a, the mass of the deposited MnO_2_ and the deposition rate over time were calculated and presented in Figure S8b. Through this, we confirmed that the amount of the deposited MnO_2_ increased with time, but the deposition rate gradually decreased. Figure 5b shows the change of the CV curves according to electroplating duration. The CV curve of the AACS NW electrode without a MnO_2_ layer showed a much smaller current density than that of the Ag/Au/MnO_2_ core–shell NW electrodes. In addition, it can be seen that the integrated area of the CV curve increases with the MnO_2_ coating time. The areal capacitances calculated from the CV curves were 0.09 mF/cm^2^ for the AACS NW electrode and 4.3, 8.7, and 12.2 mF/cm^2^ for Ag/Au/MnO_2_ core–shell NW electrodes. The areal capacitances were calculated according to $C_{a}=\frac{1}{\nu A\left(v_{f}-v_{i}\right)}\int_{v_{i}}^{v_{f}}\mathrm{IdV}$, where I is the current, $v_{i}$ and $v_{f}$ are the initial and final voltage of the CV curve, ν is the scan rate, and A is the geometric area of the electrode [26]. To identify the charge-storage mechanism of the suggested Ag/Au/MnO_2_ core–shell NW electrode, we conducted additional CV tests in various voltage scan rates, as shown in Figure S9a. The measured CV currents (at 0.4 V) and voltage scan rates were fit with the relationship of $i=av^{b}$, and the result was presented in Figure S9b. The *b* value was calculated to 0.82, indicating a mixed charge-storage mechanism including both capacitive and diffusion-controlled behaviors [27]. Subsequently, the charge and discharge experiments were performed at various current densities with a 2 min MnO_2_ coating sample, and the results are presented in Figure 5c. The quasi-symmetric triangular shape with negligible IR drop verifies the ideal capacitive characteristic of the electrode. We also studied the cyclic stability of the electrode through a repeated charge and discharge experiment at a current density of 0.2 mA cm^−2^. The areal capacitance was maintained at 91% of its initial capacitance after 500 cycles, as shown in Figure 5d. Here, the areal capacitance was calculated according to $C_{a}=\frac{i}{A}\times \frac{\Delta t}{\Delta V}$, where i/A is the current density (μA cm^−2^), Δt is the discharge time (s), and ΔV is the voltage range. In addition, the mechanical flexibility of the electrode was examined by monitoring the capacitance change under repeated bending cycles. As shown in Figure S10a, after 1000 times repeated bending with a 5.5 mm bending radius, the capacitance was maintained to 92% compared to the initial state, confirming the mechanical stability of the electrode for practical application in a flexible supercapacitor. Through the above processes, we confirmed that the Ag/Au/MnO_2_ core–shell NW electrodes have adequate capacitance, fast charge/discharge characteristics, stable cycle stability, and mechanical flexibility.

### 3.5. Electrochemical Performances of a Solid-State Flexible Supercapacitor Made with a Pair of the Ag/Au/MnO_2_ Core–Shell NW Electrodes

We fabricated an all-solid-state supercapacitor with a pair of identical Ag/Au/MnO_2_ core–shell NW electrodes and PVA/LiClO_4_ gel electrolyte. The gel electrolyte served as a separator as well. We selected the non-acidic electrolyte because we have seen that the phosphoric or sulfuric acid-based electrolytes destroyed the MnO_2_ layer. The gel electrolyte was applied to the electrodes through a dip-coating process and dried at room temperature for 4 h to evaporate moisture. After the drying process, the electrodes were attached to each other to form a solid-state supercapacitor. In this step, the electrolyte acted as an adhesive. The electrochemical performance was analyzed using a two-electrode method. 

**Figure 6:**
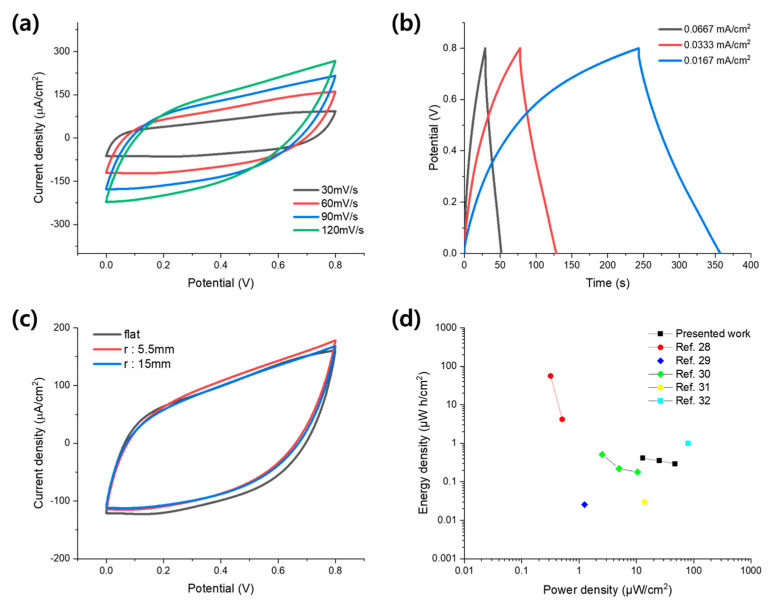
Electrochemical performances of the supercapacitor made up with a pair of the Ag/Au/MnO_2_ core−shell NW electrodes. (**a**) CV curves of a supercapacitor based on the Ag/Au/MnO_2_ (2 min) core–shell NW electrodes at different voltage scan rates. (**b**) Charge/discharge curves at different current densities. (**c**) CV curves at various bending radii with a scan rate of 60 mV/s. (**d**) Ragone plot considering the recently reported flexible supercapacitors [28,29,30,31,32].

First, a CV test was performed by varying the voltage scan rate from 30 to 120 mV/s; the results are presented in Figure 6a. Under all voltage scan rate conditions, rectangular-shaped CV curves were obtained, which shows the excellent electrostatic properties of the fabricated supercapacitor. In addition, the characteristics of a typical supercapacitor whose specific current value increased with the voltage scan rate were observed. Compared with the CV curves of the half-cell electrodes, the shape of the CV curve slightly inclined as the scan rate increased. This can be explained by the lower conductivity of the gel electrolyte than that of the liquid electrolyte. Subsequently, the charge and discharge experiments were performed, and the result are presented in Figure 6b. There was no significant IR drop in any of the curves obtained at various current densities. Furthermore, as presented in Figure 6c, the suggested Ag/Au/MnO_2_ core–shell NW-based supercapacitor exhibited good stability under various bending conditions, which is an essential factor for flexible supercapacitors. There were no noticeable changes in the CV curves measured at 60 mV/s even under the bending state with a 15 mm and 5.5 mm bending radius. It resulted from the flexibility of the NW network and the stable connections between the components: the substrate, the NW network, the MnO_2_ layer, and the gel electrolyte. The mechanical stability was further examined by monitoring the capacitance change under repeated bending conditions and the result is presented in Figure S10b. After 500 times repeated bending cycles, the capacitance was maintained at 94%, confirming the mechanical stability and flexibility of the suggested Ag/Au/MnO_2_ core–shell NW network electrodes. Finally, we evaluated the performance of the fabricated flexible supercapacitor through a Ragone plot, as shown in Figure 6d. The energy and power density were calculated according to $E=\frac{1}{2}\times C_{a}\times \frac{{\left(\Delta V\right)}^{2}}{3600}$ and $P=\frac{E}{\Delta t}\times 3600$, respectively [26]. From the Ragone plot, it was confirmed that the suggested Ag/Au/MnO_2_ flexible supercapacitor was able to store 0.41 μWh/cm^2^ at 12.9 μW/cm^2^, which is comparable (or even better) to some of the previously reported flexible supercapacitors using various materials such as the MnO_2_/Au (4.2 μWh/cm^2^ at 0.51 μW/cm^2^), the GO/CNT (25.5 nWh/cm^2^ at 1.25 μW/cm^2^), the rGO/Cu-MOF (0.51 μWh/cm^2^ at 2.54 μW/cm^2^), the ZnO NW (0.03 μWh/cm^2^ at 14 μW/cm^2^), and the MnO_2_/Ti3C2Tx MXene (0.7 μWh/cm^2^ at 80.0 μW/cm^2^). Moreover, it is worth mentioning that the method reported here has significant advantages as it is facile and could be used with other various electrode materials.

## 4. Conclusions

Herein, we have described the facile fabrication process of AACS NWs using a simple solution process with a suppressed galvanic replacement reaction. The thin Au layer on the Ag NW surface acted as a protective layer, enhancing the electrochemical stability of the Ag NWs and provided an additional electrical path that allowed direct electroplating of the MnO_2_ nanostructure on the Ag NW network electrode. The prepared Ag/Au/MnO_2_ core–shell NW electrode exhibited excellent capacitive properties (4.3–12.2 mF/cm^2^) and a fast charge–discharge rate in Na_2_SO_4_ aqueous electrolyte, which was mainly due to the adequate electrical connection between the NW network and MnO_2_ nanostructures. Moreover, a symmetric solid-state supercapacitor was prepared to examine the mechanical stability of the electrode. Despite the repeated mechanical deformations, the Ag/Au/MnO_2_ core–shell NW electrode-based supercapacitor maintained its performance (94% areal capacity retention after 500 cycles), which is a critical factor in the practical application of flexible supercapacitors. Our results suggested that the AACS NW network electrode could serve as a mechanically and electrochemically stable current collector for a flexible supercapacitor. Considering that the other capacitive materials such as RuO_2_ or conducting polymers can be directly electroplated on the suggested electrode, it could widely expand its application in flexible and transparent energy storage devices, including supercapacitors and batteries.

## Data Availability

Not applicable.

## Supplementary Materials

The following are available online at https://www.mdpi.com/article/10.3390/ma14143934/s1, Figure S1. (a) Porous Au nanotubes resulted from the galvanic replacement of Ag NW. (b) Ag NW aggregation resulted from excessive injection rate of Au precursor solution during Au coating process; Figure S2. (a) TEM image and measured diameter values of the Ag NW. (b) TEM image of the AACS NWs for measuring the diameters; Figure S3. TEM-EDX mapping images of the AACS NWs; Figure S4. (a) TEM image of the pristine Ag NW. (b) TEM-EDX line profile of the pristine Ag NW. (c) TEM image of the AACS NW. (d) TEM-EDX line profile of the AACS NW; Figure S5. The result of the repeated bending test of the Ag NW and AACS NW network electrode; Figure S6. (a) Comparison of the electrical resistance of the electrodes before, and after the electroplating process. (b) Light transmittance of the Ag NW and AACS NW before, and after 1min electroplating. (c) Digital image of the Ag NW, AACS NW and Ag/Au/MnO_2_ NW electrodes. (d) CV curves of the electrodes after the electroplating of the MnO_2_; Figure S7. XRD spectrum of the Ag/Au/MnO_2_ electrode; Figure S8. (a) Dependence of the current density on the deposition time during electroplating process. (b) The estimated weight of the electrodeposited MnO_2_ and the deposition rate as functions of the deposition time; Figure S9. (a) CV curves of the AACS NW electrode obtained with various scan rate. (b) Log plot between CV currents (at 0.4 V) and scan rates, based on the relationship of $i=av^{b}$; Figure S10. (a) The bending test result for the Ag/Au/MnO_2_ electrode as a half-cell supercapacitor. (b) The result of the same bending test for the all solid-state supercapacitor fabricated with a pair of Ag/Au/MnO_2_ electrodes.

## References

[1] Chen F., Wan P., Xu H., Sun X. (2017). Flexible Transparent Supercapacitors Based on Hierarchical Nanocomposite Films. ACS Appl. Mater. Interfaces.

[2] González A., Goikolea E., Barrena J.A., Mysyk R. (2016). Review on supercapacitors: Technologies and materials. Renew. Sustain. Energy Rev..

[3] Raza W., Ali F., Raza N., Luo Y., Kim K.-H., Yang J., Kumar S., Mehmood A., Kwon E.E. (2018). Recent advancements in supercapacitor technology. Nano Energy.

[4] Kumar K.S., Choudhary N., Jung Y., Thomas J. (2018). Recent Advances in Two-Dimensional Nanomaterials for Supercapacitor Electrode Applications. ACS Energy Lett..

[5] Dong L., Xu C., Li Y., Huang Z.-H., Kang F., Yang Q.-H., Zhao X. (2016). Flexible electrodes and supercapacitors for wearable energy storage: A review by category. J. Mater. Chem. A.

[6] Han Y., Dai L. (2019). Conducting Polymers for Flexible Supercapacitors. Macromol. Chem. Phys..

[7] Liang X., Long G., Fu C., Pang M., Xi Y., Li J., Han W., Wei G., Ji Y. (2018). High performance all-solid-state flexible supercapacitor for wearable storage device application. Chem. Eng. J..

[8] Cheng T., Zhang Y.-Z., Zhang J.-D., Lai W.-Y., Huang W. (2016). High-performance free-standing PEDOT:PSS electrodes for flexible and transparent all-solid-state supercapacitors. J. Mater. Chem. A.

[9] Liu X., Li D., Chen X., Lai W.-Y., Huang W. (2018). Highly Transparent and Flexible All-Solid-State Supercapacitors Based on Ultralong Silver Nanowire Conductive Networks. ACS Appl. Mater. Interfaces.

[10] Lee D., Lee H., Ahn Y., Lee Y. (2015). High-performance flexible transparent conductive film based on graphene/AgNW/graphene sandwich structure. Carbon.

[11] Xu F., Zhu Y. (2012). Highly Conductive and Stretchable Silver Nanowire Conductors. Adv. Mater..

[12] Selzer F., Weiß N., Bormann L., Sachse C., Gaponik N., Müller-Meskamp L., Eychmüller A., Leo K. (2014). Highly conductive silver nanowire networks by organic matrix assisted low-temperature fusing. Org. Electron..

[13] McLellan K., Yoon Y., Leung S.N., Ko S.H. (2020). Recent Progress in Transparent Conductors Based on Nanomaterials: Advancements and Challenges. Adv. Mater. Technol..

[14] Giovanni M., Pumera M. (2012). Size Dependant Electrochemical Behavior of Silver Nanoparticles with Sizes of 10, 20, 40, 80 and 107 nm. Electroanalysis.

[15] Khan A., Nguyen V.H., Muñoz-Rojas D., Aghazadehchors S., Jiménez C., Nguyen N.D., Bellet D. (2018). Stability Enhancement of Silver Nanowire Networks with Conformal ZnO Coatings Deposited by Atmospheric Pressure Spatial Atomic Layer Deposition. ACS Appl. Mater. Interfaces.

[16] Lee H., Hong S., Lee J., Suh Y.D., Kwon J., Moon H., Kim H., Yeo J., Ko S.H. (2016). Highly Stretchable and Transparent Supercapacitor by Ag–Au Core–Shell Nanowire Network with High Electrochemical Stability. ACS Appl. Mater. Interfaces.

[17] Rauda I.E., Augustyn V., Dunn B., Tolbert S.H. (2013). Enhancing Pseudocapacitive Charge Storage in Polymer Templated Mesoporous Materials. Acc. Chem. Res..

[18] Choi C., Ashby D.S., Butts D.M., DeBlock R.H., Wei Q., Lau J., Dunn B. (2020). Achieving high energy density and high power density with pseudocapacitive materials. Nat. Rev. Mater..

[19] Lu Q., Chen J.G., Xiao J.Q. (2013). Nanostructured Electrodes for High-Performance Pseudocapacitors. Angew. Chem. Int. Ed..

[20] Kang J., Hirata A., Kang L., Zhang X., Hou Y., Chen L., Li C., Fujita T., Akagi K., Chen M. (2013). Enhanced Supercapacitor Performance of MnO_2_ by Atomic Doping. Angew. Chem. Int. Ed..

[21] Yu Z., Duong B., Abbitt D., Thomas J. (2013). Highly Ordered MnO_2_ Nanopillars for Enhanced Supercapacitor Performance. Adv. Mater..

[22] Huang M., Li F., Dong F., Zhang Y.X., Zhang L. (2015). MnO_2_-based nanostructures for high-performance supercapacitors. J. Mater. Chem. A.

[23] Yang Y., Liu J., Fu Z.-W., Qin D. (2014). Galvanic Replacement-Free Deposition of Au on Ag for Core–Shell Nanocubes with Enhanced Chemical Stability and SERS Activity. J. Am. Chem. Soc..

[24] Lee Y., Chae S., Park H., Kim J., Jeong S.-H. (2020). Stretchable and transparent supercapacitors based on extremely long MnO_2_/Au nanofiber networks. Chem. Eng. J..

[25] Guo C., Ma H., Zhang Q., Li M., Jiang H., Chen C., Wang S., Min D. (2020). Nano MnO_2_ Radially Grown on Lignin-Based Carbon Fiber by One-Step Solution Reaction for Supercapacitors with High Performance. Nanomaterials.

[26] Zhao W., Jiang M., Wang W., Liu S., Huang W., Zhao Q. (2021). Flexible Transparent Supercapacitors: Materials and Devices. Adv. Funct. Mater..

[27] Song Y., Pan Q., Lv H., Yang D., Qin Z., Zhang M., Sun X., Liu X. (2021). Ammonium-Ion Storage Using Electrodeposited Manganese Oxides. Angew. Chem..

[28] Moon H., Lee H., Kwon J., Suh Y.D., Kim D.K., Ha I., Yeo J., Hong S., Ko S.H. (2017). Ag/Au/Polypyrrole Core-shell Nanowire Network for Transparent, Stretchable and Flexible Supercapacitor in Wearable Energy Devices. Sci. Rep..

[29] Peng H., Zhong Y., Zhang X., He Y., Wang G. (2018). Percolating Film of Pillared Graphene Layer Integrated with Silver Nanowire Network for Transparent and Flexible Supercapacitors. Langmuir.

[30] Wang Y.-F., Yang S.-Y., Yue Y., Bian S.-W. (2020). Conductive copper-based metal-organic framework nanowire arrays grown on graphene fibers for flexible all-solid-state supercapacitors. J. Alloy. Compd..

[31] Singh S.B., Kshetri T., Singh T.I., Kim N.H., Lee J.H. (2019). Embedded PEDOT: PSS/AgNFs network flexible transparent electrode for solid-state supercapacitor. Chem. Eng. J..

[32] Zhang C., Anasori B., Seral-Ascaso A., Park S.-H., McEvoy N., Shmeliov A., Duesberg G.S., Coleman J., Gogotsi Y., Nicolosi V. (2017). Transparent, Flexible, and Conductive 2D Titanium Carbide (MXene) Films with High Volumetric Capacitance. Adv. Mater..

