# Supplementary material for: Electrodeposition of the MnO2 on the Ag/Au Core–Shell Nanowire and Its Application to the Flexible Supercapacitor
Source: Materials (Basel). 2021 Jul 14;14(14):3934. doi: 10.3390/ma14143934 (PMC8303347; doi:10.3390/ma14143934)
Supplement: Supplementary file 1 [file materials-14-03934-s001.zip › materials-1281663-supplementary.pdf]

materials

MDPI

Supporting Information

Electrodeposition of the MnO_2 on the Ag/Au Core-Shell Nanowire and Its Application to the Flexible Supercapacitor

Wonbin Seo [1], Dongwoo Kim [1], Shihyeong Kim [2] and Habeom Lee [1,*]

[1] School of Mechanical Engineering, Pusan National University, 2, Busandaehag-ro, 63 Beon-gil, Ge-umjeong-gu, Busan, 46241, Korea; e-mail@e-mail.com
[2] Technical Textile & Materials R&D Group, Korea Institute of Industrial Technology, Ansan, Gyeonggi-do 15588, Korea 2; e-mail@e-mail.com
* Correspondence: Correspondence: hblee@pusan.ac.kr; Tel.: +82-51-510-2891

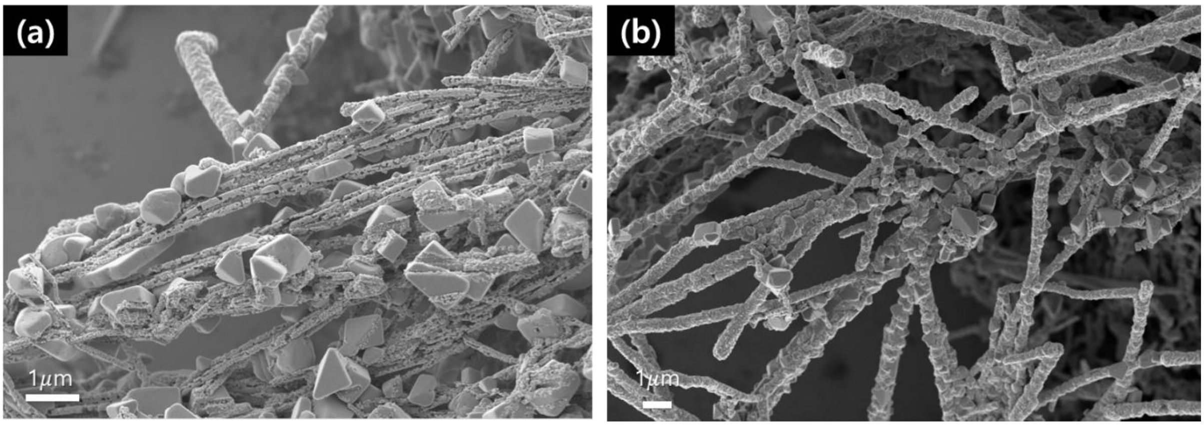

Figure S1. (**a**) Porous Au nanotubes resulted from the galvanic replacement of Ag NW. In the galvanic replacement, the Au atoms replaced the Ag atoms of Ag NW to form porous Au nanotubes. (**b**) Ag NW aggregation resulted from excessive injection rate of Au precursor solution during Au coating process. To prevent the NW aggregation, we used an injection speed of 50 μL/min or less.

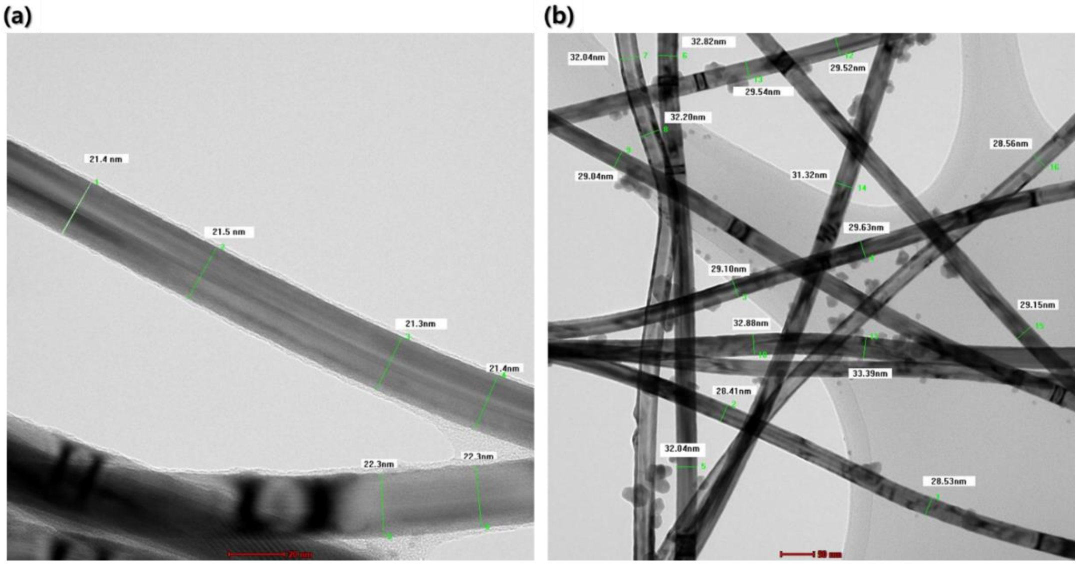

Figure S2. Diameter measurement through the TEM analysis. (**a**) TEM image and measured diameter values of the Ag NW. The values matched the information (21±3 nm) provided by the Ag NW supplier, Flexio Co. (**b**) TEM image of the AACS NWs for measuring the diameters. The averaged diameter of the AACS NW was 31±2 nm. Comparing the diameters of Ag NW and AACS NW, we confirmed that the thickness of the Au shell coated on the Ag NW surface was ~ 5nm.

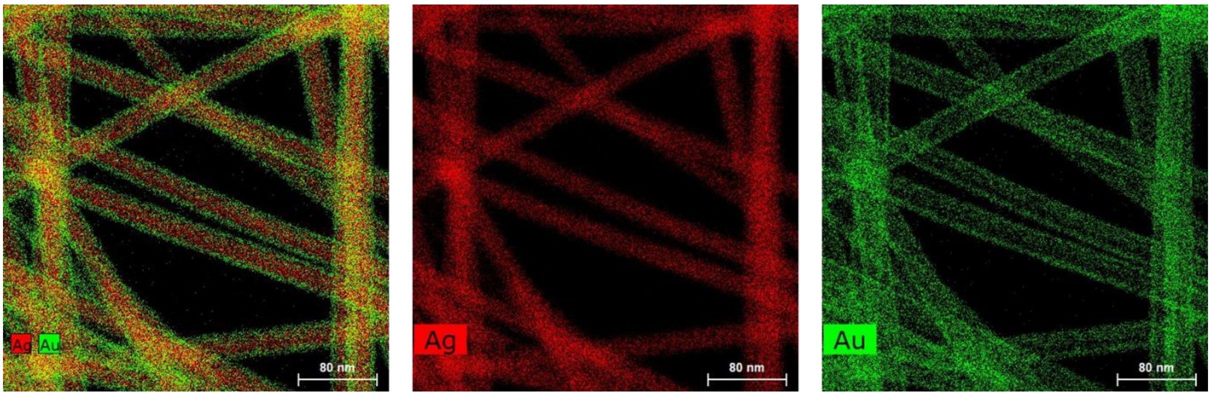

Figure S3. TEM-EDX mapping images of the AACS NWs. In the mapping image it can be seen that the Au shells covered the Ag NW surface.

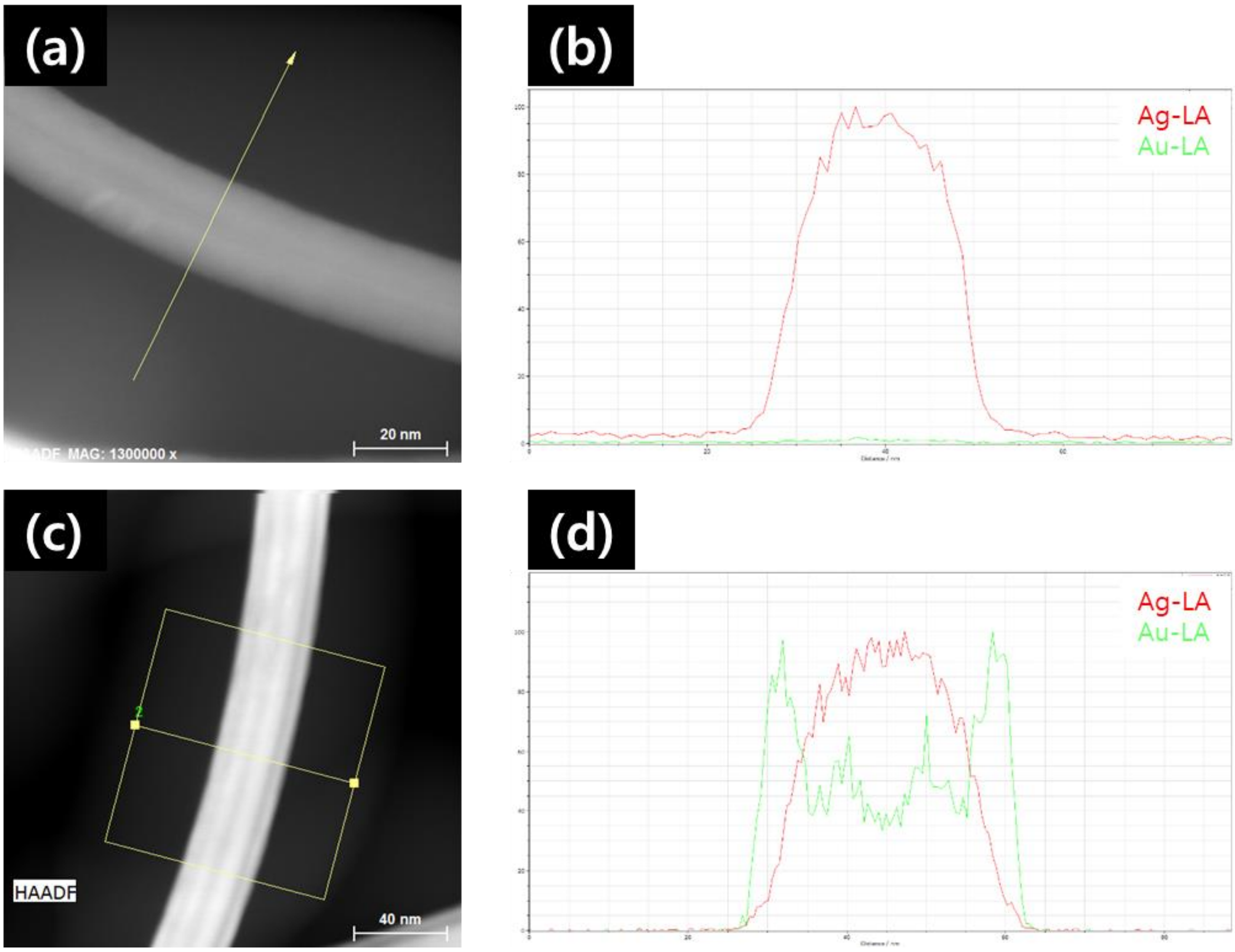

Figure S4. (**a**) TEM image of the pristine Ag NW. (**b**) TEM-EDX line profile of the pristine Ag NW. (**c**) TEM image of the AACS NW. (**d**) TEM-EDX line profile of the AACS NW. TEM-EDX line profiles were measured following the yellow line of the TEM images. From the EDX analysis, we confirmed that the brightness contrast of the AACS NW TEM image in Fig 2(d) resulted from the Au layer on the Ag NW surface.

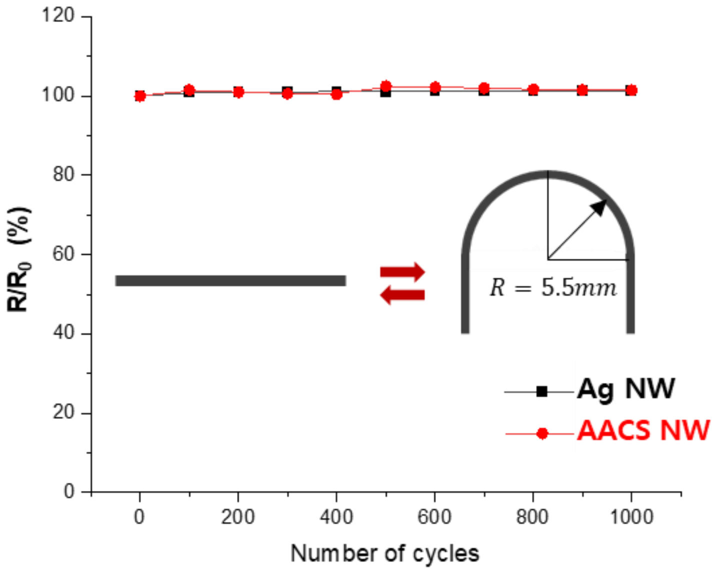

Figure S5. The result of the repeated bending test of the Ag NW and AACS NW network electrode. Both electrodes stably maintained their electrical conductivity during the 1000 times bending cycles with a bending radius of 5.5 mm.

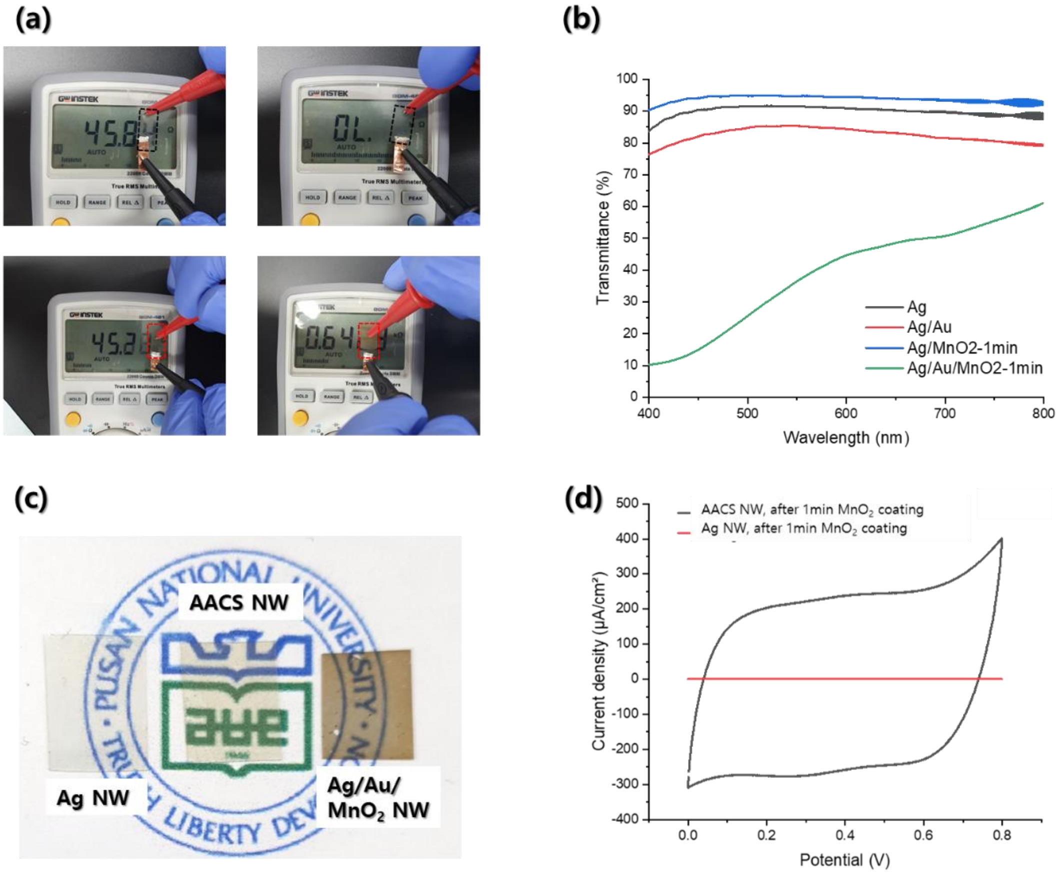

Figure S6. (**a**) Comparison of the electrical resistance of the electrodes before, and after the electroplating process. The upper images show the Ag NW network electrode, and the lower images show the AACS NW electrodes. The left images are the electrodes before electroplating, and the right images are the electrodes after electroplating. Unlike the AACS NW electrode, the Ag NW electrodes lost its electrical conductivity during the electroplating process. (**b**) Light transmittance of the Ag NW and AACS NW before, and after 1min electroplating. The transmittance was measured in visible wave length range. (**c**) Digital image of the Ag NW, AACS NW and Ag/Au/MnO_2 NW electrodes. (**d**) CV curves of the electrodes after the electroplating of the MnO_2.

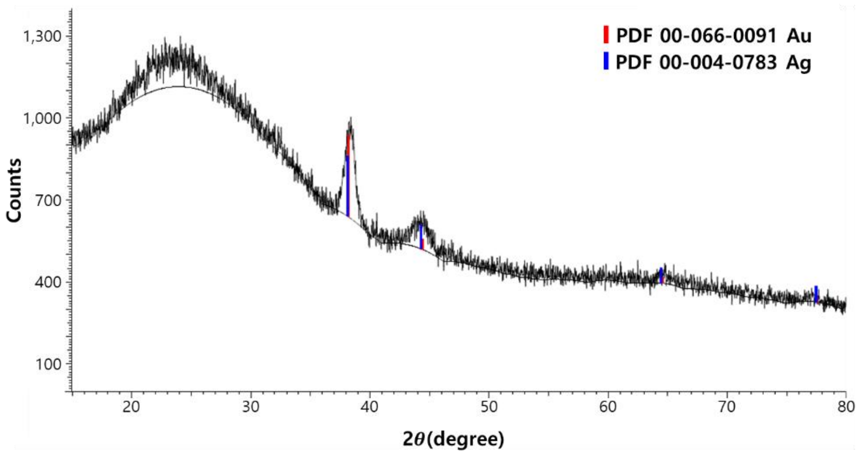

Figure S7. XRD spectrum of the Ag/Au/MnO_2 electrode. In the XRD analysis, we could not observe any peaks from the MnO_2. Rather than, only the peaks corresponding to the Au and the Ag were detected.

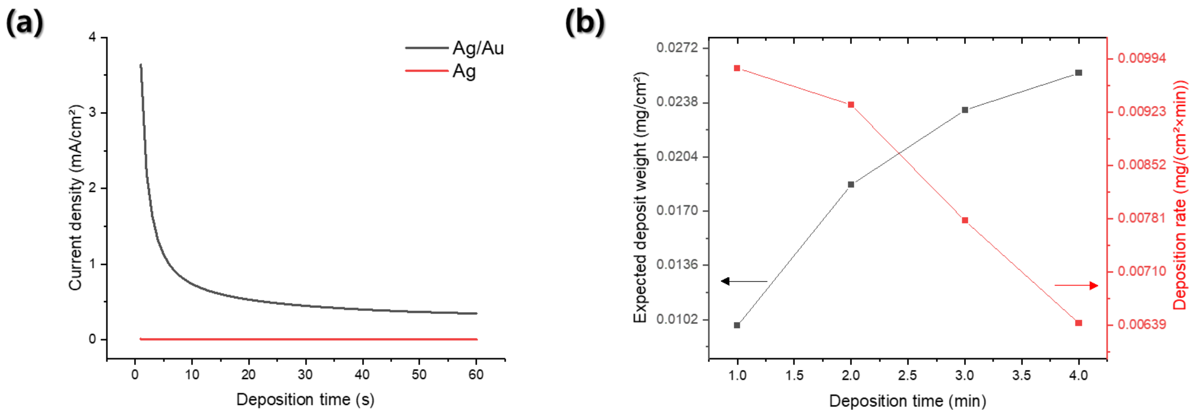

Figure S8. (**a**) Dependence of the current density on the deposition time during electroplating process. The rapid decrease of the current density implies that the semiconductive MnO_2 started to cover the surface of the AACS NWs from the beginning of the electroplating process. (**b**) The estimated weight of the electrodeposited MnO_2 and the deposition rate as functions of the deposition time. The estimated mass of the deposited MnO_2 was calculated using below equations.

$$\mathrm{Mn(NO_3)_2 + 2e^- \rightarrow MnO_2 + 2NO_2{}^-}.$$

$$m = \frac{Q \times M}{F \times z}$$

$$\mathrm{Q} = \int_0^t Idt$$

where m is the mass of MnO_2 deposited in grams, Q is the total electric charge passing through the cell in coulombs, F is the Faraday constant (*96485 C/mol*), M is the molar mass of MnO_2 in grams per mole, and z is electron transfer per mole, I is the time dependent current of the MnO_2 deposition process, and t is the total time of deposition. The calculated deposition mass per area increased with time from 0.016 mg/cm^2 at 1 min to 0.041 mg/cm^2 at 4 min. However, the deposition rate gradually decreased over time.

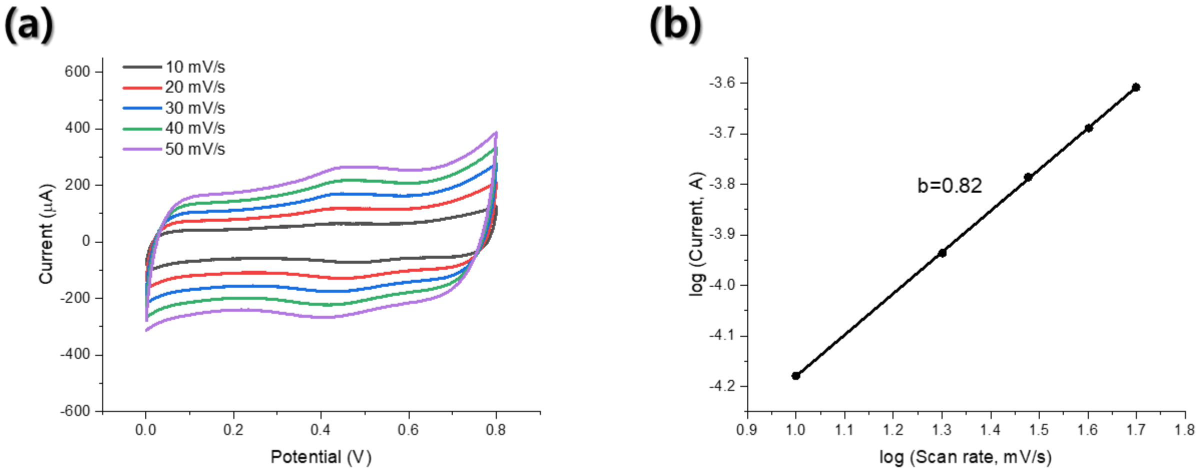

Figure S9. (**a**) CV curves of the AACS NW electrode obtained with various scan rate. (**b**) Log plot between CV currents (at 0.4 V) and scan rates, based on the relationship of $i = av^b$, where i is the CV current, v is the scan rate, and a and b are the fitting parameters. The "b" value was calculated to 0.82.

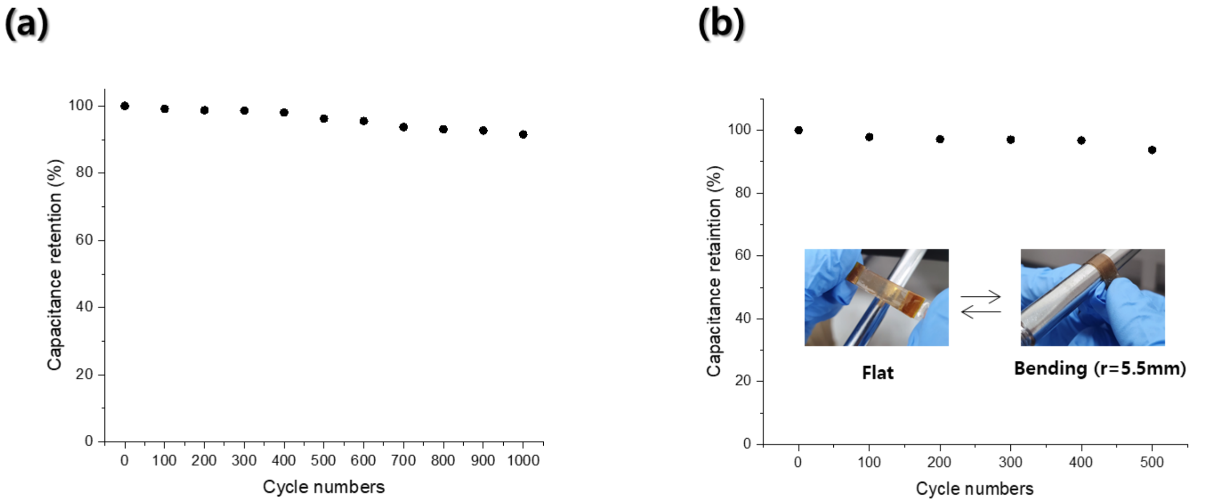

Figure S10. The results of the repeated bending tests. (**a**) The bending test result for the Ag/Au/MnO_2 electrode as a half-cell supercapacitor. The bending radius was set as 5.5 mm. The areal capacitance was measured every 100 bending cycles with a CV test using the three-electrode method. (**b**) The result of the same bending test for the all solid-state supercapacitor fabricated with a pair of Ag/Au/MnO_2 electrodes. The areal capacitance was measured every 100 bending cycles with a CV test using the two-electrode method.
